# Performance and risk in the Brazilian banking industry

**DOI:** 10.1016/j.heliyon.2021.e06524

**Published:** 2021-03-28

**Authors:** Francisco Javier Sáez-Fernández, Andrés J. Picazo-Tadeo, Ignacio Jiménez-Hernández

**Affiliations:** a Department of Spanish and International Economics, University of Granada, Spain; b Department of Applied Economics II, University of Valencia; and Joint Research Group INTECO, Spain

**Keywords:** Brazilian banking industry, Commercial and investment banks, Data Envelopment Analysis, Directional distance functions, Performance, Risk

## Abstract

This paper assesses the technical performance of Brazilian banks while accounting for risk, which is considered as an undesirable outcome of banking. To this end, frontier techniques based on Data Envelopment Analysis and directional distance functions are applied to a sample of 124 banks and data for the six-year period 2014–19. Our main finding is that the Brazilian banking industry could notably increase its production of conventional outputs without additional input usage and while maintaining the same levels of risk. Besides, investment banks are found to be more efficient than commercial banks mainly because of their superior managerial performance.

## 1 Introduction

The banking industry plays a key role in modern economies. Banks are financial intermediaries that gather deposits and other liabilities from savers and transfer them to borrowers in the form of loans and other financial assets. They share certain functions with financial markets, such as resource allocation, reducing credit risk, intermediating maturity differences, or bearing interest rate and exchange rate risk. Furthermore, the banking industry faces three main sources of risk: credit risk, operational risk, and market risk. Banks' productive process as financial intermediaries and the risk inherent to banking should not be considered individually, but rather jointly analysed. In this regard, existing research shows that risk exerts a major influence on both the level and variability of banks' performance; besides, these effects differ over time and across countries (Sun and Chang, 2011).

In this research, we assess the technical efficiency of Brazilian banks while accounting for risk, which is considered as an undesirable outcome of banking. Besides, we study the difference in performance between groups of banks and the sources of these differences, particularly distinguishing commercial banks from investment banks. In doing so, we use non-parametric Data Envelopment Analysis (DEA) techniques on a sample of 124 Brazilian banks, and data for years 2014–19. Regarding the contributions of the paper, while the analysis of efficiency in the banking industry has received a great deal of attention in recent decades, approaches accounting for risk as an undesirable by-product of banking—as our paper does—are much scarcer. Another contribution is the assessment of the differences in performance between commercial and investment banks and the sources of those differences, considering that any increase in the production of conventional outputs is limited not only by resource availability but also by the need to keep risk under control. Moreover, as far as we know, no previous studies have taken risk into account when examining the efficiency of Brazilian banks.

The Brazilian banking industry is the largest in the Latin American and Caribbean region. Furthermore, the banking system has historically played an important role as financial intermediary in Brazil given the lack of development of financial markets, and particularly the corporate bond market (Staub et al., 2010). The banking industry in this country has undergone important structural transformation in recent decades, which makes an analysis of its performance particularly interesting.

As in other major Latin American emerging countries, the 1990s in Brazil were characterized by rapid economic development largely motivated by the Washington Consensus. Moreover, privatizations, foreign direct investment incentives, financial liberalization, price stabilization and other reforms contributed to the integration of Brazil's domestic financial market into international financial markets (De Paula, 2011). The new regulation of the financial system enacted in 1988 allowed banks to offer different financial services, universalizing their business. In June 1994, the Brazilian government instituted a monetary reform aimed at stabilizing prices—the so-called Real Plan*—*which led to a profound reformulation of the banking sector (Almeida and Divino, 2015), and a notable increase in credit. The banking industry also witnessed a number of mergers and acquisitions involving both domestic and foreign banks (Baer and Nazmi, 2000). In August 1996, the Central Bank of Brazil launched the PROES (Program of Incentives for the Reduction of the State's Participation in Banking Activities), which was aimed at restructuring public banks; as a result of this programme only 12 of the 32 public banks that existed in 1994 remained operative in 2012 (Wolters et al., 2014).

Following this Introduction, Section 2 reviews existing literature on banking performance and risk; Section 3 explains the methodology; Section 4 describes the sample and the data; Section 5 presents and discusses the results; finally, Section 6 concludes.

## 2 Background

Previous literature has addressed the study of performance in the banking industry from different angles and using a range of methodological approaches. Surveys in the field include Berger (2007), which reviews the extant literature on the sources of differences in performance, including measurement method and a number of bank, market, and regulatory features; Paradi and Zhu (2013), which conducts a survey on bank branch efficiency and performance research in 24 countries or economic areas carried out with DEA; and, more recently, Aiello and Bonanno (2018), which reviews the empirical literature on banking efficiency by conducting a meta-regression analysis from 120 papers published over the period 2000–14. Without aiming to be exhaustive, papers focused on the Brazilian banking industry include: Ceretta and Niederauer (2001), Silva (2001), Silva and Neto (2002), Becker et al. (2003), Macedo et al. (2005), Tabak et al. (2005), Ghilardi (2006), Souza et al. (2006), Chabalgoity et al. (2007), Périco et al. (2008), Ruiz et al. (2008), Souza et al. (2008), Souza and Macedo (2009), Staub et al. (2010), Tecles and Tabak (2010), Wanke and Barros (2014), Wanke et al. (2015), Périco et al. (2016), De Freitas Branco et al. (2017), Gomes et al. (2017), and Henriques et al. (2018).

Regarding the contributions and main findings of these papers, Silva (2001) analysed X-efficiency of Brazilian banks in the period 1994-99—after the implementation of the Real Plan—discovering a certain variability in efficiency, mostly stemming from public banks. Tecles and Tabak (2010) studied the post-privatization period 2000–07 finding that the negative profits reported by many banks in Brazil were closely related to prior privatization waves; these authors also suggested that large banks are the most cost and profit efficient, which supports the concentration process observed in previous years. Staub et al. (2010) found that the efficiency of the Brazilian banking industry in 2000–07 was lower than that of European and North American banks; and that state-owned banks in Brazil were the most cost efficient. Wanke and Barros (2014) analysed the efficiency of major Brazilian banks and its drivers in 2012. The results brought to light the heterogeneity of the banking industry in Brazil; also, mergers and acquisitions were found to be the main driver of both productive and cost efficiency. Henriques et al. (2018) evaluated the efficiency of 37 Brazilian banks in 2012–16 with DEA, and the causes of inefficiency. The authors found large inefficiencies that are slightly more related to technical issues than to the scale of operations; they also recommended fostering mergers and acquisitions as a strategy to improve performance.

The structure of the banking industry worldwide and the relationships among its players changed substantially from 2008, as a result of several regulatory reforms oriented to addressing the moral hazard problem arising from banks aiming to increase returns by taking increasingly risky positions in the securities markets. In September 2010, the Basel Committee for Banking Supervision passed new regulations for capital requirements—Basel III. Back in 1988, Basel I had tackled the impact of banks' capital regulations on their risk-taking performance. These regulatory changes stimulated a line of research focused on explaining the relationship between risk, capital and performance in banking. The theoretical models underpinning empirical work are mainly grounded on three hypotheses: i) the *bad management* hypothesis, which holds that managing risk requires the use of resources that could otherwise be dedicated to other productive activities (Williams, 2004); ii) the *bad luck* hypothesis, which emphasizes the role of external triggers instead of managers' skills (Berger and DeYoung, 1997); and iii) the *moral hazard* hypothesis, which posits that managers tend to take on more risk when banks have lower levels of capital or they are less profit efficient (Jeitschko and Jeung, 2005).

Kwan and Eisenbeis (1997) analysed the interrelationships among banks' interest rate and credit risk-taking, capitalization, and efficiency; their results support the moral hazard hypothesis. Williams (2004) examined the intertemporal relationships between loan loss provision, efficiency and capitalization for European banks between 1990 and 1998, with the findings supporting the bad management hypothesis. In this regard, managers who engage in skimping behaviour reduce the use of bank resources that are oriented to monitoring the lending business; this influences the quality of loans and cost efficiency because bank managers face a trade-off between short-term operating costs and future loan quality. Fiordelisi et al. (2011) examined the inter-temporal link between bank efficiency, capital and risk in European commercial banks in 1997–2005. The results suggest that lower cost and revenue efficiency causes higher bank risk. Tan and Floros (2013) studied the relations among bank efficiency, capital and risk in Chinese commercial banks over the period 2003–09. Their findings suggest that there is a positive significant relationship between risk and efficiency, while the relationship between risk and capitalization is negative and also significant.

In a different framework, Hughes and Mester (1998) found empirical evidence that the managers of US banks use more labour and physical capital in order to ensure better risk management and capital preservation, according to the bad management hypothesis. Altunbas et al. (2007) studied the relation between capital, risk and efficiency of European banks in the period 1992–2000, finding no empirical evidence of a relationship between efficiency and bank risk-taking. Recently, Colesnic et al. (2019) analysed the effect of risk on Middle East banks' efficiency levels before and after the financial crisis of 2008. In doing so, they defined an indicator of banks' risk efficiency which accounts for the inefficiency due to risk abatement cost—i.e., risk is considered as an undesirable or bad output in the banking production function (see Assaf et al., 2013). The empirical findings suggest that large banks' risk management was more flexible during the financial crisis; most notably, the authors advise that omitting risk may lead to biased estimates of banks' efficiency.

## 3 Methodology

The study of performance in banking has been addressed using different methodological approaches, notable among which are Data Envelopment Analysis (DEA) and Stochastic Frontier Analysis (SFA). Both of these approaches have their pros and cons. DEA is a non-parametric technique based on mathematical programming developed by Charnes et al. (1978), which has been employed in hundreds of empirical papers on efficiency assessment (for a recent survey see Emrouznejad and Yang, 2018; see Paradi and Zhu, 2013 for those focused on banking). Conversely, SFA is an approach simultaneously proposed by Aigner et al. (1977) and Meeusen and Van den Broeck (1977), which is grounded in the estimation of parametric production or cost functions. According to Hjalmarsson et al. (1996, p.304), ‘…*the choice between different approaches* [to performance assessment] *must be based on trade-offs concerning the purpose of the study, type of data, technology characteristics, etc.*’.

In our paper, we have decided in favour of non-parametric DEA primarily due to its flexibility. In this regard, DEA does not require a particular functional form to be established for either the technology or the distribution of efficiency, which greatly facilitates the task of accounting for risk as an undesirable output of banking (Jiménez-Hernández et al., 2019b). Instead, this technique allows a surface to be built over a set of observed data on productive units*—*banks in our case study*—*representing the best observed practices. All the productive units in the sample are then projected onto this technological frontier, yielding an indicator of performance (see details in Cooper et al., 2007). As noted by Färe et al. (1994, p.11), this approach constitutes ‘...*an elegant way of simultaneously constructing frontier technology from data and calculating the distance to that frontier for individual observations or activities*’. Furthermore, a notable feature of DEA—which is particularly relevant for the purpose of our research—is that it enables the computation of a range of measures of performance that might represent the preferences of researchers or managers; in the case of this paper, how the production of conventional outputs could be increased without employing additional resources and also maintaining risk at observed levels.

### 3.1 Using DEA to assess performance in banking while accounting for risk

Let us assume that we observe a sample of b = 1,...,B banks using a set of N inputs $\text{x}\in R_{+}^{\text{N}}$ to obtain a vector of M good outputs represented by $\text{y}\in R_{+}^{\text{M}}$. Transforming inputs into good outputs necessarily generates a certain level of risk, which is represented by a set of H variables $\text{r}\in R_{+}^{\text{H}}$; moreover, risk is considered as an undesirable or *bad* output from banking.

The technology that models the transformation of inputs into good outputs and *bad* outputs (risk) is represented by:

(1) T=[(y,r,x)| xcanproduce(y, r)]

It is assumed that the technology satisfies the axioms proposed by Shephard (1970), including possibility of inaction, no free lunch, strong disposability of inputs and good outputs, and convexity. Inputs, good outputs and *bad* outputs are all considered to be non-negative. Furthermore, in order to model the joint production of good outputs and *bad* outputs two further axioms are needed: null-jointness and weak disposability of outputs, both good and *bad*.

*Null-jointness* models the idea that good outputs and *bad* outputs (risk) are jointly produced (Shephard and Färe, 1974). Put simply, if banks produce a positive amount of good outputs, some risk will also unavoidably be assumed. In formal terms:

(2) If (y,r, x)∈Tandr=0,theny=0

*Weak disposability of outputs—*desirable and undesirable*—*means that reducing risk is not free, but it has an opportunity cost that can be assessed in terms of a reduction in the potential amount of good outputs produced. This is because resources such as employees or physical capital that could otherwise be dedicated to producing good outputs ought to be diverted to activities devoted to reducing risk. Formally:

(3) If (y,r,x)∈Tand0≤α≤1,then(αy,αr,x)∈T 

The relative position of each bank in the sample with respect to the technology defined in expression (1) can be assessed, in terms of an indicator of performance, using directional distance functions (DDFs). The more general formulation of the DDF is (Färe and Grosskopf, 2000):

(4) $$\vec{\text{DDF}}=\left[\text{y},\text{r},\text{x};\text{g}=\left({\text{g}}_{\text{y}},-{\text{g}}_{\text{r}},-{\text{g}}_{\text{x}}\right)\right]=\text{Sup}\left[\text{β}|\left(\text{y}+\text{β}{\text{g}}_{\text{y}},\text{r}-\text{β}{\text{g}}_{\text{r}},\text{x}-\text{β}{\text{g}}_{\text{x}}\right)\in \text{T}\right]$$

This DDF generalizes both Shephard's input and output distance functions (Shephard 1970) by jointly modelling good outputs, risk and inputs. It thereby provides, in the most general setting, a measure of the extent to which the good outputs could be increased in a direction ${\text{g}}_{\text{y}}$, while risk and inputs are respectively reduced in directions $-{\text{g}}_{\text{x}}$ and $-{\text{g}}_{\text{r}}$. By construction, DDFs are lower bounded to zero. Other properties are detailed in Chambers et al. (1998).

Let us now consider that we are interested in assessing banks' performance in the presence of risk as the maximum proportional attainable increase in the good outputs while maintaining the same level of risk and input usage^1^; i.e., the good output-oriented approach. In this scenario, the DDF of expression (4) becomes:

(5) $$\vec{\text{DDF}}=\left[\text{y},\text{r},\text{x};\text{g}=\left(\text{y},0,0\right)\right]=\text{Sup}\left[\text{β}|\text{⟨}(1+\text{β})\text{y},\text{r},\text{x}\text{⟩}\in \text{T}\right]$$

where g=(y,0,0) is the direction vector that represents our preferences on how to measure banks' performance.

By way of example, a computed score for the DDF from expression (5)*—*parameter β*—*for a given bank of, let us say, 0.25 would mean that by behaving efficiently this bank could proportionally increase its good outputs by 25% without increasing risk and/or input usage. In terms of the expression (1+β), the potential or efficient level of the good outputs would be 1.25 times their observed level.

Alternatively, the technology can be characterized by assuming that *bad* outputs (risk) are strongly (or freely) disposable, which allows us to compute an indicator of the opportunity cost of reducing risk at the bank level. Strong disposability of the *bad* outputs can be formalized as:

(6) If (y,r,x)∈Tand0≤α≤1,then(y,αr,x)∈T

The assumption of *strong disposability* of *bad* outputs means, as proposed by Färe et al. (1989), that reducing *bads* is costless; in simpler words, risk can be reduced at no cost. Moreover, strong disposability disrupts the physical link between good outputs and risk, rendering the null-jointness hypothesis unnecessary. In the *real world*, this would make little sense since there is always an association between good outputs and risk in banking; e.g., no loans can be made without assuming some risk, no matter how small. Accordingly, strong disposability needs to be understood in terms of costs, as Färe et al. (1989) themselves emphasized.

By comparing the DDFs of expression (5) computed with respect to technologies characterized by both weak (${\vec{\text{DDF}}}^{\text{W}}$) and strong disposability (${\vec{\text{DDF}}}^{\text{S}}$), we can compute an indicator of the opportunity cost of reducing risk at the bank level, expressed in terms of potential good output losses. Formally, for good output m, this indicator is:

(7) $$\text{Good}\;\text{output}\;\text{loss}\;{\text{y}}_{\text{m}}={\text{y}}_{\text{m}}\left({\vec{\text{DDF}}}^{\text{S}}-{\vec{\text{DDF}}}^{\text{W}}\right),$$

This indicator measures potential losses of good outputs due to weak disposability, and by construction is equal to or larger than zero. A positive value indicates that weak disposability of *bads* is reducing the potential increases in the good outputs; i.e., reducing risk requires the use of resources that otherwise could be dedicated to producing the good outputs.

**Figure 1:**
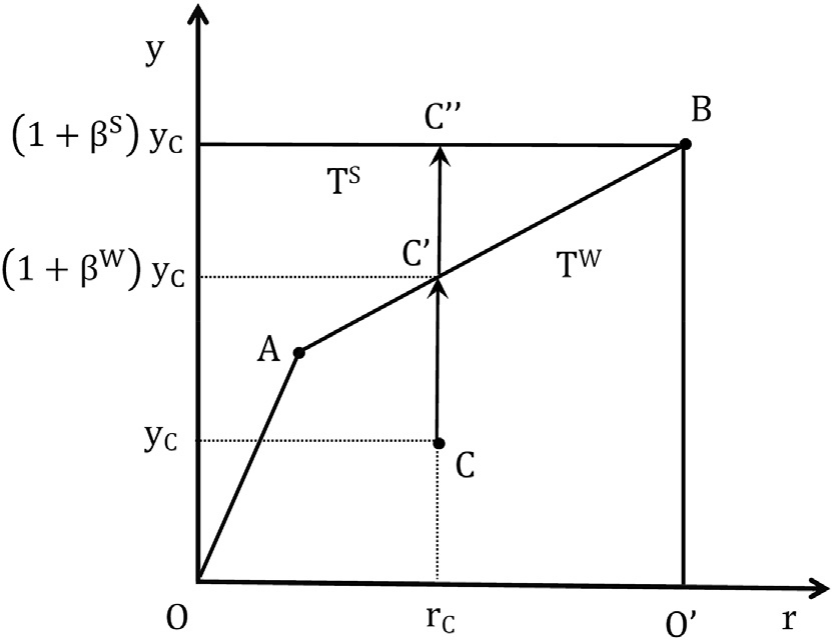
Strong and weak disposability of outputs.

Figure 1 graphically depicts the technologies under weak and strong disposability, and the assessment of potential output losses. For the sake of simplicity, let us consider that we observe banks A, B and C, which all use the same vector of inputs to produce one good output (y) and one *bad* output (risk) (r). The technology that satisfies the assumptions of weak disposability and null-jointness (T^W^) is bounded by OABO′, whereas the technology where the good and *bad* outputs are strongly (freely) disposable (T^S^) is bounded by OO’B and the horizontal segment that goes from B until the vertical good-output axis. Furthermore, bank C is inefficient with respect to both technologies, as it is producing in an inner point of the output set.

Projecting the observed production plan of bank C toward the frontier of T^S^ in the good output direction yields*—*according to expression (5)*—*point C’’. This means that when reducing risk is assumed to be costless, the potential good output of bank C would be $\left(1+{\text{β}}^{\text{S}}\right)$ times its observed level if it acted efficiently; in other words, the good output could be increased by a proportion of β^S^, a quantity equivalent to the segment CC’’. On the other hand, when it is assumed that reducing risk is costly, the potential increase in the good output when point C is projected onto the frontier of T^W^ comes down to the proportion β^W^; or by the corresponding quantity CC’. This reduction of the *efficient* good output due to the weak disposability of risk is just what expression (7) measures; i.e., the loss of potential good output given by C’C’’.

In practice, computing the DDFs involved in our assessment of banking efficiency under the weak disposability axiom (T^W^) and a direction that increases the good outputs, while maintaining productive resources and risk at observed levels, entails solving the following program for each bank b´:

(8) $${\vec{\text{DDF}}}^{\text{W}}\left[\text{y},\text{r},\text{x};\text{g}=\left(\text{y},0,0\right)\right]={\text{Max }}_{{\text{z}}^{\text{b}},{\text{μ}}^{\text{b}},{\text{β}}_{\text{b'}}^{\text{W}}}{\text{β}}_{\text{b'}}^{\text{W}}$$

Subject to:

$$\begin{array}{l} \left(1+{\text{β}}_{\text{b'}}^{\text{W}}\right){\text{y}}_{\text{mb'}}\leq \overset{\text{B}}{\underset{\text{b}=1}{\sum }}{\text{z}}_{\text{b}}{\text{y}}_{\text{mb}}\;\text{m }=\;1\text{,}\ldots \text{,M}\;\left(\text{i}\right)\\ {\text{r}}_{\text{hb'}}=\overset{\text{B}}{\underset{\text{b}=1}{\sum }}{\text{z}}_{\text{b}}{\text{r}}_{\text{hb}}\;\text{h }=\;1\text{,}\ldots \text{,H}\;\left(\text{ii}\right)\\ {\text{x}}_{\text{nb'}}\geq \overset{\text{B}}{\underset{\text{b}=1}{\sum }}\left({\text{z}}_{\text{b}}+{\text{μ}}_{\text{b}}\right){\text{x}}_{\text{nb}}\;\text{n }=\;1\text{,}\ldots \text{,N}\;\left(\text{iii}\right)\\ \overset{\text{B}}{\underset{\text{b}=1}{\sum }}\left({\text{z}}_{\text{b}}+{\text{μ}}_{\text{b}}\right)=1\;\left(\text{iv}\right)\\ {\text{z}}_{\text{b}},{\text{μ}}_{\text{b}}\geq 0\;\text{b }=\;1\text{,}\ldots \text{,B}\;\left(\text{v}\right) \end{array}$$

In program (8) variable returns to scale (VRS) are imposed through restriction (iv) (Banker et al., 1984).^2^ Nonetheless, as noted by Zago and Donceli (2011, p.542), the standard DEA-based specification grounded on VRS prevents the technology from satisfying weak disposability of both good and *bad* outputs (risk), thus hindering the usage of our risk-augmented model of performance. In order to overcome this weakness, we have used the approach proposed by Kuosmanen (2005) (see also Kuosmanen and Podinovski, 2009), which allows performance assessment with VRS and weak disposability; accordingly, μ_b_ denotes the so-called *scale effect*, and z_b_ stands for the *efficient effect* (further technical details are in Kuosmanen, 2005, p.1079–80).

Likewise, computing the DDFs against a technology with strong disposability (T^S^) and the abovementioned direction vector that increases the good outputs, while maintaining inputs and risk, requires solving the following program, also for each bank b´:

(9) $${\vec{\text{DDF}}}^{\text{S}}\left[\text{y},\text{r},\text{x};\text{g}=\left(\text{y},0,0\right)\right]={\text{Max }}_{{\text{λ}}^{\text{b}},{\text{β}}_{\text{b'}}^{\text{S}}}{\text{β}}_{\text{b'}}^{\text{S}}$$

Subject to:

$$\begin{array}{l} \left(1+{\text{β}}_{\text{b'}}^{\text{S}}\right){\text{y}}_{\text{mb'}}\leq \overset{\text{B}}{\underset{\text{b}=1}{\sum }}{\text{λ}}_{\text{b}}{\text{y}}_{\text{mb}}\;\text{m }=\;1\text{,}\ldots \text{,M}\;\left(\text{i}\right)\\ {\text{r}}_{\text{hb'}}\leq \overset{\text{B}}{\underset{\text{b}=1}{\sum }}{\text{λ}}_{\text{b}}{\text{r}}_{\text{hb}}\;\text{h }=\;1\text{,}\ldots \text{,H}\;\left(\text{ii}\right)\\ {\text{x}}_{\text{nb'}}\geq \overset{\text{B}}{\underset{\text{b}=1}{\sum }}{\text{λ}}_{\text{b}}{\text{x}}_{\text{nb}}\;\text{n }=\;1\text{,}\ldots \text{,N}\;\left(\text{iii}\right)\\ \overset{\text{B}}{\underset{\text{b}=1}{\sum }}{\text{λ}}_{\text{b}}=1\;\left(\text{iv}\right)\\ {\text{λ}}_{\text{b}}\geq 0\;\text{b }=\;1\text{,}\ldots \text{,B}\;\left(\text{v}\right) \end{array}$$

with ${\text{λ}}_{\text{b}}$ standing for the elements of the so-called intensities vector.

### 3.2 The metatechnology approach

One crucial assumption in Section 3.1 is that all banks in the sample share a common technology. However, certain banks may have no access to some production plans within the common technology due to regulations or other physical, social or economic factors in their environment. In such cases, the question arises as to whether their inefficiency is due to poor management or rather to the restrictions imposed by these environmental factors. The metafrontier approach by O'Donnell et al. (2008) allows some light to be shed on this question.

Let us define the *metatechnology* (MT) as the set of all feasible combinations of inputs, good outputs and risk available to the banking industry according to the state-of-knowledge, as defined in expression (1). It is assumed that the metatechnology also satisfies the axioms of null-jointness and weak disposability of outputs. The *directional metadistance function* (DMDF) can be computed against the metatechnology according to expression (5) in the particular case of assuming a direction that increases the good output while keeping inputs and risk the same. These DMDFs are assumed to fulfil the same properties as DDFs.

Furthermore, let us consider that the banks in our sample can be grouped into g = 1,…,G categories, according to criteria relating to features of their operating environment. As already noted, the central issue is that belonging to a given group might prevent banks from having access to the entire set of feasible production plans in the metatechnology. That said, the technology of group g under weak disposability^3^ representing the set of feasible production plans available to banks in that group is:

(10) $${\text{T}}^{\text{Wg}}=\left[\left(\text{y},\text{r},\text{x}\right)|\text{x}\;\text{can}\;\text{be}\;\text{used}\;\text{by}\;\text{banks}\;\text{in}\;\text{group}\;\text{g}\;\text{to}\;\text{produce}\left(\text{y},\text{r}\right)\right]$$

Having defined the technology for group g, the DDF that allows us to compute the potential increase in the good outputs while maintaining the same level of inputs and risk with respect to the technology of that group is:

(11) $${\vec{\text{DDF}}}^{\text{g}}=\left[\text{y},\text{r},\text{x};\text{g}=\left(\text{y},0,0\right)\right]=\text{Sup}\left[{\text{β}}^{\text{Wg}}|\text{⟨}(1+{\text{β}}^{\text{Wg}})\text{y},\text{r},\text{x}\text{⟩}\in {\text{T}}^{\text{Wg}}\right]$$

By way of example, a computed score for the expression $\left(1+{\text{β}}^{\text{Wg}}\right)$ for a bank belonging to group g of, let us say, 1.1 would indicate that it could increase its good outputs by 10%, while maintaining the same level of risk and with no additional input usage, when compared to best observed practices within its own group. The DDF computed with respect to the technology of group g will be, by construction, always equal to or lower than the DMDF relative to the metatechnology; i.e., the potential of a bank to expand its good outputs when it is compared to the metatechnology will always be greater than that obtained when it is compared to banks in its own group.

Comparing the DMDFs obtained with respect to the metatechnology with the DDFs computed relative to the group frontiers allows us to define the metatechnology ratio for group g as:^4^

(12) $${\text{Metatechnology ratio}}^{\text{g}}=\frac{1+\vec{\text{DMDF}}}{1+{\vec{\text{DDF}}}^{\text{g}}}=\frac{\left(1+{\text{β}}^{\text{W}}\right)}{\left(1+{\text{β}}^{\text{Wg}}\right)}$$

Expression (12) measures how close the technological frontier of group g is from the metafrontier, assessed in a direction that increases the good outputs while keeping both input usage and risk the same. As pointed out by O'Donnell et al. (2008, p.237), this approach allows for a suitable decomposition of *overall performance*, assessed with respect to the metafrontier, into i) *managerial performance*, measured with respect to the group technology; and ii) *group performance*, measured by the metatechnology ratio. In formal terms:

(13) $$\text{Overall}\;\text{performance}={\text{Managerial performance}}^{\text{g}}\times {\text{Group performance}}^{\text{g}}$$

For illustrative purposes, an overall performance score of 1.5 for a given bank would mean that it could increase its good outputs by 50% for given inputs and risk. This score could be the result of, let us say: i) a managerial performance score of 1.2, meaning that using the best practices available to banks in its own group, the good outputs could be increased by 20%; and ii) a group performance score of 1.25, which means that, once the *efficient* levels of the good outputs against the group technology have been reached, an additional increase of 25% over those levels could be achieved if this bank used the best practices in the entire set of production plans available to the banking industry, given by the metatechnology.

**Figure 2:**
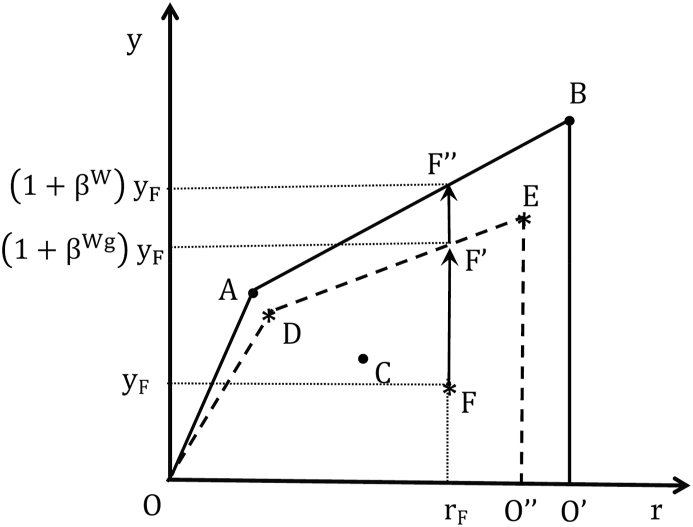
Performance, distance/metadistance functions and the metatechnology ratio.

Figure 2 graphically depicts this decomposition. Let us assume that banks in our sample can be classified into two groups: banks A, B and C (represented by dots) belong to group 1, while banks D, E and F (denoted by asterisks) belong to group 2. Efficient banks A and B and their convex combinations shape the technological frontier of group 1, which is given by the segment OABO’; likewise, the technological frontier of group 2 is ODEO”, which is shaped by efficient banks D and E and their convex combinations. It is also assumed that the metatechnology, or technological frontier for the whole sample, coincides with the frontier of group 1.

Let us now assess the performance of bank F belonging to group 2. Projecting its production plan onto the technological frontier of the group to which it belongs in, let us say, a North direction, yields point F’; i.e., by efficiently using the technology available to banks in its group bank F could attain a potential good output $\left(1+{\text{β}}^{\text{Wg}}\right)$ times its observed level; in other words, the good output could be increased by a proportion of ${\text{β}}^{\text{Wg}}$ (*managerial performance*). Similarly, projecting onto the metafrontier yields point F″, indicating that if bank F had access to the entire set of production plans in the metatechnology, it could achieve a good output of $\left(1+{\text{β}}^{\text{W}}\right)$ times its observed level; or a proportional increase of ${\text{β}}^{\text{W}}$ (*overall performance*). The difference between the good outputs at points F′ and F″ is a measure of the effect on performance of belonging to a particular group (*group performance*).

Using DEA, the metadistance functions involved in the calculation of the technology ratio of expression (12) can be directly computed from program (8) using the entire sample of banks; likewise, computing the distances with respect to group technologies requires running program (8) using only the sample of banks belonging to each group.

## 4 The production function in banking: data, variables and sample

### 4.1 The production function in the banking industry

The existing literature has considered two main approaches to characterize the production function in the banking industry: the production approach and the intermediation approach. The former considers banks as firms that produce deposits and loan account services from traditional inputs; e.g., physical capital and labour. Conversely, the intermediation approach regards banks as financial intermediaries between savers and investors, which secure deposits and other funds and use them to produce different types of loans and other assets.

In this research, we use the intermediation approach (Sealey and Lindley, 1977), which is the most habitual in analyses of banking performance. Besides, we follow the asset approach for the selection of inputs and outputs, which considers banks as financial intermediaries only between liability holders and those who receive bank funds. Loans and other assets are considered bank outputs, whereas deposits and other liabilities play the role of inputs in the intermediation process (Berger and Humphrey, 1992).

Based on the abovementioned arguments, the inputs included in our characterization of the banking production function are i) staff expenses, to account for labour, and ii) non-earning assets, as a proxy of physical capital; in addition, we incorporate three financial inputs iii) equity; iv) customer deposits; and v) market liabilities, calculated as the sum of bank deposits, derivative financial instruments and trading liabilities. It should be noted that we have included a wide range of bank liabilities in order to account for the inputs of both commercial banks—which usually have a bigger role in the retail market—and investment banks—which are traditionally more market-oriented. On the other hand, conventional or good outputs are i) gross loans and ii) securities.

Finally, our variable accounting for the risk intrinsic to banking activity is the standard deviation of the return on assets (ROA), computed at the bank level over a five-year window. Furthermore, in order to perform a robustness analysis of our results we also consider the standard deviation of the return on equity (ROE) as an alternative measure of risk (see Liu et al., 2012). In both cases, a larger deviation represents higher risk.

### 4.2 Data and sample

The empirical analysis carried out in this research is based on data from Moody's Analytics BankFocus, a database that includes information about 55,700 banks worldwide. It is managed by the Bureau van Dijk and Moody's Investors Service and the data come from a mixture of annual reports, information delivered by banks and regulatory sources. The dataset includes accounting and financial statistics that are highly suitable for making comparisons between banks, and also offer good coverage of the Brazilian banking industry.

According to this dataset, a total of 165 banks were operating in Brazil in the year 2019. The industry is highly concentrated as the five largest banks account for about 80% of total banking assets. Furthermore, the market is largely dominated by domestic institutions, both private and public. In fact, the five largest banks in terms of assets—Banco do Brasil, Itaú, Caixa Economica Federal (CEF), Bradesco and BNDES—are domestic; moreover, CEF and BNDES are majority state-owned. It is also worth highlighting the notable role in the Brazilian banking industry played by banks providing several banking services, including retail services, investment banking services, and brokerage services.

To build our sample, we have used yearly data from 2014 to 2019 inclusive for Brazilian banks in the BankFocus dataset.^5^ At the time of writing this paper, 2019 was the last year for which data were available. Moreover, given the serious lack of data for some banks prior to 2014, it was considered advisable not to extend the sample any further back as its representativeness could be affected. It is important, however, to highlight that the use of data from several years is not primarily intended to analyse the time dimension of performance in the Brazilian banking industry, but to overcome a common limitation of DEA. This approach suffers from a lack of discrimination power when there is a small number of observations relative to the number of inputs and outputs; and this could be the case with our empirical application^6^ (see Dyson et al., 2001, for details). Including more observations in the sample by considering the time dimension of the data is expected to greatly improve the discrimination power of our DEA-based models (Cooper et al., 2007; Jiménez-Hernández et al., 2019a). That said, it also entails the assumption that no significant technical change occurred over the period 2014–2019, which, in our view, is fairly plausible.

Thus, after removing banks with missing data for some of the variables defined in Section 4.1, and detecting and eliminating outliers by means of scatter plots and the *trimmean* function applied to 5% of the observations, our final dataset includes information on 124 Brazilian banks over the abovementioned six-year period. All bank and year observations have been pooled into a single sample. Moreover, given that data for some of the banks are not available in particular years, our final dataset includes a total of 543 observations. This final sample represents 97% of the total assets of Brazilian banks included in the Moody's Analytics BankFocus dataset for the period analysed; and 67% of the banks—a percentage that goes up to 75% in the year 2019, for which more data are available. Table 1 provides some descriptive statistics for the variables that represent the production process in banking. The high standard deviations of some of these variables brings to light the large size differences among the banks operating in the Brazilian banking industry.

**Table 1:** Sample description (*constant 2019 $US million*).

	Mean	Standard deviation
Inputs		
Staff expenses	334	1,196
Non-earning assets	3,779	13,894
Equity	1,804	5,788
Customer deposits	6,179	23,792
Market liabilities	1,406	4,642
Good outputs		
Loans	8,922	33,008
Securities	4,929	18,893
Risk		
Standard deviation of ROA	1.91	3.54
Standard deviation of ROE	8.87	16.94

Source: Authors' elaboration from Moody's Analytics BankFocus.

## 5 Results and discussion

The results for the technical efficiency of Brazilian banks in the sample under both weak and strong disposability assumptions, in addition to potential good output losses, are in Table 2. These results have been obtained from programs (8) and (9) with the standard deviation of ROA computed over a five-year window, which includes the year to which the observation belongs and the four previous years, as a measure of risk; and from expression (7) for the potential output loss.^7^ Above all, the low technical efficiency of the banking industry in Brazil stands out. In the scenario where it is assumed that reducing risk requires the use of resources that otherwise could be devoted to producing good outputs, banks in the sample could increase their loans and securities by a proportion of 65.1%, on average, without further usage of inputs and maintaining the same level of risk. The low efficiency of Brazilian banks has also been reported in previous studies such as Tabak et al. (2005), Souza et al. (2006) and, more recently, Henriques et al. (2018). However, the contribution of our research is that technical efficiency is evaluated while accounting for risk, which allows a more accurate assessment of performance.

**Table 2:** Estimates of technical efficiency (*1 best*) and potential output loss.

	Mean	Standard deviation
Weak disposability assumption $\left(1+{\text{β}}^{\text{W}}\right)$	1.651	0.780
Strong disposability assumption $\left(1+{\text{β}}^{\text{S}}\right)$	1.691	0.809
Potential good output loss $\left({\text{β}}^{\text{S}}-{\text{β}}^{\text{W}}\right)$	0.040	0.157

Source: Authors' elaboration.

Conversely, when it is assumed that reducing risk is a costless activity, the average proportional potential increase in the good outputs that could be achieved without consuming additional inputs is 69.1%, regardless of the level of risk. This finding clearly shows how reducing risk has a sizeable opportunity cost measured as a lower feasible expansion of the good outputs, thus supporting the bad management hypothesis, proposed by Williams (2004). The extent of the potential output loss due to weak disposability of risk can be interpreted as a reduction of 4 percentage points in the efficient level of the good outputs, on average.^8^

Several papers focused on the analysis of performance in the Brazilian banking industry have assessed the differences in efficiency between groups of banks. According to the information provided by the Moody's Analytics BankFocus dataset, in our sample of 543 observations, 427 correspond to commercial banks whereas 116 are categorized as investment banks.^9^ Moreover, 240 observations are identified as belonging to domestic banks, while 196 correspond to foreign banks; finally, 445 observations belong to private banks and only 12 to public ones.^10^

Table 3 displays the estimated scores of technical efficiency by groups of banks. It is worth highlighting that these scores of technical efficiency correspond to the scenario of weak disposability, which represents the *real world* where reducing risk consumes productive resources. At first glance, investment banks (score of 1.563, indicating that by behaving efficiently banks in this group could proportionally increase their good outputs by an average of 56.3%, without additional input usage and also maintaining the same level of risk) seem to perform better than commercial banks (score of 1.675); domestic banks (1.626) also achieve better performance than foreign ones (1.687); and finally, public banks (1.198) seem to be more technically efficient than private ones (1.693). These results are in line with Souza et al. (2006), which found Brazilian domestic banks to be more efficient than foreign ones, and Wanke and Barros (2014), which concluded that public ownership correlates with larger efficiency. However, given the large standard deviations of our efficiency scores, the question arises as to whether the abovementioned differences are statistically significant.

**Table 3:** Estimates of technical efficiency (*1 best*) by groups of banks under the weak disposability assumption $\left(1+{\text{β}}^{\text{W}}\right)$.

	Mean	Standard deviation
Commercial banks	1.675	0.785
Investment banks	1.563	0.753
Domestic banks	1.626	0.690
Foreign banks	1.687	0.860
Private banks	1.693	0.794
Public banks	1.198	0.293

Source: Authors' elaboration.

In order to further investigate this issue, we employ the Kolmogorov-Smirnov test of the equality of distributions, and the Mann–Whitney test that checks the hypothesis that two samples come from the same population (see Conover, 1999). In addition, we apply the Simar–Zelenyuk–Li test (Simar and Zelenyuk, 2006), which was explicitly designed for testing the equality of distributions of technical efficiency scores calculated using DEA. In essence, the algorithm of this test is based on the computation and bootstrapping of the Li statistic (Li, 1996) using DEA estimates, where scores equal to unity have been previously smoothed by adding a small noise component. The results are in Table 4. All three tests suggest that the difference of performance between commercial and investment banks is statistically significant. Besides, the results from both the Kolmogorov-Smirnov and Mann–Whitney tests point to the lack of significance of the difference in performance between domestic and foreign banks, although the Simar–Zelenyuk–Li test suggests weak significance, only at 10%. Finally, the technical efficiency of Brazilian private banks is statistically different from that of public ones, according to the results of the Kolmogorov-Smirnov and Mann–Whitney tests, but not the Simar–Zelenyuk–Li test.

**Table 4:** Statistical significance of the differences in technical efficiency.(1)

	Kolmogorov-Smirnov test (*KS-statistic*)(2)	Mann-Whitney test (*Z-statistic*)(3)	Simar-Zelenyuk-Li test (*Li-statistic*)(4)
Commercial *versus* investment	0.168 (0.010)^∗∗∗^	-2.128 (0.033)^∗∗^	2.676 (0.003)^∗∗∗^
Domestic *versus* foreign	0.105 (0.164)	-0.182 (0.855)	1,363 (0.086)^∗^
Private *versus* public	0.359 (0.073)^∗^	-2.140 (0.032)^∗∗^	0.188 (0.425)

Source: Authors' elaboration.

∗ means significance at 10%.

(1) *P-values* are in parentheses; ^∗∗∗^ and ^∗∗^ mean significance at 1% and 5%, respectively.

(2) Null hypothesis: the two samples have the same distribution; the exact *p-values* are computed.

(3) Null hypothesis: the two samples are drawn from the same population. *Z-statistic* adjusted for ties.

(4) Original estimates are smoothed using the algorithm II in Simar and Zelenyuk (2006, p.508).

Given the aforesaid results, we can state with a high degree of confidence that Brazilian commercial banks perform differently from investment banks. However, reasonable doubts arise concerning the differences in performance between domestic and foreign banks, on the one hand, and between private and public banks, on the other. In the first case, only the Simar–Zelenyuk–Li test finds the difference to be (weakly) statistically significant. In the second, the reason is twofold: the Simar–Zelenyuk–Li test does not support the statistical significance of this difference, and this test is specifically designed for efficiency scores such as those calculated in this research; and there are only 12 observations in the group of public banks—belonging to the 2 banks observed over the period 2014-2019—which seriously limits the representativeness in this group. Accordingly, the following question arises: Why do Brazilian investment banks perform better than commercial ones?

### 5.1 Commercial *versus* investment banks: managerial or group performance?

In Section 3.1 it was assumed that all banks in the sample share a common technology, regardless of the group to which they belong. However, in practice it might be the case that, due to particular environmental circumstances, some banks do not have access to the complete set of production plans available in the common technology. Thus, the question arises as to whether their inefficiency is due to poor management or to the technological restrictions imposed by such environmental factors. In this regard, the metafrontier approach developed in Section 3.2. helps us to further investigate the differences in performance between Brazilian commercial and investment banks.

Table 5 displays the results of decomposing the overall technical efficiency of commercial and investment banks as the result of managerial efficiency and group efficiency.^11^ As already pointed out, the averages of overall efficiency for commercial and investment banks are 1.675 and 1.563, respectively, with the difference being statistically significant. Furthermore, when commercial banks are compared to best observed practices in their group, their managerial efficiency is, on average, 1.503; this score indicates that if all managers of commercial banks in the sample performed as efficiently as the best managers in the group, the good outputs could be increased by an average of 50.3% while maintaining input usage and risk the same. Average managerial efficiency for investment banks is 1.224, suggesting a potential increase in the good outputs of 22.4%. Although these figures cannot be directly compared to each other since they have been computed relative to different frontiers—i.e., the technologies of commercial banks and investment banks, respectively—they allow us to assert that, on average, the managers of investment banks are operating closer to their technological frontier than commercial bank managers are to theirs.

**Table 5:** Managerial efficiency *versus* group efficiency (*1 best*).

	Commercial banks		Investment banks	
	Mean	Standard deviation	Mean	Standard deviation
Overall efficiency $\left(1+{\text{β}}^{\text{W}}\right)$	1.675	0.785	1.563	0.753
Managerial efficiency $\left(1+{\text{β}}^{\text{Wg}}\right)$	1.503	0.638	1.224	0.398
Group efficiency $\left(1+{\text{β}}^{\text{W}}\right)/\left(1+{\text{β}}^{\text{Wg}}\right)$	1.109	0.227	1.250	0.367

Source: Authors' elaboration.

Comparing the scores of technical efficiency relative to both the metafrontier and the group frontiers allows the calculation of the metatechnology ratio for all banks in the sample, or group efficiency. As explained in Section 3.2, these ratios evaluate how close the technologies of investment banks and commercial banks are to the metatechnology or common technology, thus permitting an assessment of which technology is more efficient. According to our results, the average group efficiency of commercial banks is 1.109; this score indicates that even after reaching the level of the good outputs enabled by the best practices available to managers of commercial banks, a further increase of 10.9% over this level could still be achieved if the banks had access to the entire set of production plans in the metatechnology. Average group efficiency for investment banks is 1.250, pointing to an additional potential increase in the good outputs of 25%.

But is the difference in group efficiency between commercial and investment banks statistically significant? According to the results from the Kolmogorov-Smirnov and Mann-Whitney tests reported in Table 6, it is significant. Nonetheless, it is not significant according to the Simar-Zelenyuk-Li test, which, let us once again recall, is specifically designed for the type of efficiency scores computed in this research. Hence, we cannot robustly demonstrate that the technologies of Brazilian commercial and investment banks are different. In this regard, there have been historical technological differences between commercial banks and investment banks, mostly due to regulation and different operational capabilities. However, banking legislation has become less restrictive over the years, with a global trend towards the universal banking model; this shift might have narrowed the technological differences between commercial and investment banks.

**Table 6:** Statistical significance of the differences in group efficiency.(1)

	Kolmogorov-Smirnov test (*KS-statistic*)(2)	Mann-Whitney test (*Z-statistic*)(3)	Simar-Zelenyuk-Li test (*Li-statistic*)(4)
Commercial *versus* investment	0.190 (0.002)***	2.871 (0.004)***	0.371 (0.355)

Source: Authors' elaboration.

(1) *P-values* are in parentheses; *** mean significance at 1%.

(2) Null hypothesis: the two samples have the same distribution; the exact *p-values* are computed.

(3) Null hypothesis: the two samples are drawn from the same population. *Z-statistic* adjusted for ties.

(4) Original estimates are smoothed using the algorithm II in Simar and Zelenyuk (2006, p.508).

All in all, our results suggest that when technical efficiency is assessed while accounting for risk, Brazilian investment banks are more efficient than commercial banks. The reason is that investment banks have better managers—in the sense that they operate closer to their technological frontier representing best practices in the group—since no significant differences are found in the technology used by the two groups of banks.

Finally, the sensitivity of our findings to changes in the variable used to measure risk has been assessed by using the standard deviation of ROE instead of the deviation of ROA. This alternative scenario yields the same conclusions as those set out in the previous paragraphs. The numerical results are in the Appendix.

## 6 Conclusions

The study of performance in the banking industry has a deep-rooted tradition in the field of Economics. Since the 1980s, a bourgeoning literature has arisen devoted to analysing banks' performance from diverse approaches and perspectives. However, the related literature is less prolific when it comes to analysing the relationship between performance and risk. Although this matter has received increasing attention from researchers in the last two decades, there are still gaps that require further investigation.

This paper assesses the technical efficiency of Brazilian banks while accounting for risk. Our theoretical background is the *bad management* hypothesis posed by Williams (2004), which stresses that the management of risk requires the use of resources that could otherwise be dedicated to other productive activities; accordingly, efficiency is not independent of the risk levels that banks assume. Risk is proxied by the standard deviation of the return on assets (ROA). Furthermore, risk-conditioned scores of technical efficiency are calculated under two key assumptions, namely, that banking intermediation services cannot be produced without assuming a certain level of risk, whether high or low; and that risk can be treated as an undesirable or *bad* output from banking to be minimized. Regarding the methodology, non-parametric frontier techniques based on Data Envelopment Analysis (DEA) are applied to a sample of 124 Brazilian banks and data for the period 2014–19.

In line with some previous studies, we find that Brazilian banks are, on average, rather inefficient, although cross-bank differences are important. Moreover, an opportunity cost of maintaining risk at observed levels is found, which provides empirical support to the *bad management* hypothesis, and shows how assessing banking efficiency without properly accounting for risk could lead to biased results. Furthermore, we find robust statistical evidence that Brazilian investment banks perform better than commercial ones. Besides, domestic and foreign banks do not exhibit significant differences in performance; this result—which is in line with the findings reported by Sáez-Fernández et al. (2015) for the Latin American and Caribbean banking industry—is perhaps a consequence of the successful adaptation of Brazilian domestic banks to the process of external opening and liberalization that began in the 1990s with the Washington Consensus.

Additionally, we find that investment banks outperform commercial ones because their managers are operating closer to the best practices available to them than commercial bank managers are to theirs; accordingly, no robust empirical evidence is found that the technologies of the two groups of banks are different. Put more simply, investment banks are more technically efficient because of their superior managerial performance. Although we have no clear-cut explanations for this finding, it could be related to the greater degree of specialization of investment banks, which would generate comparative advantages. Investment banks may also have better qualified managers, which would seem essential given the type of clients they serve and the set of banking services offered.

It is our hope that the results from this research will provide bank managers and regulators of the banking industry in Brazil with sound information that can help them to improve both management and regulatory policies. In this regard, up to the best of our knowledge, we contribute the first assessment of Brazilian banks' performance accounting for risk, an intrinsic feature of financial activity that is displaying a growing trend in this turbulent new stage of the globalization era. Beyond this contribution, our research is not without its limitations, which may however pave the way for future work. We consider that further investigation is needed into the risk-conditioned performance of the Brazilian banking industry, using different methodological approaches and concepts of efficiency and risk. Moreover, a more in-depth analysis—i.e., using larger samples and more powerful statistical tests—of the possible differences in technology between groups of entities, as well as the causes of investment banks' superior performance in managerial efficiency, would also be welcome.

## Declarations

### Author contribution statement

Ignacio JIMÉNEZ-HERNÁNDEZ: Conceived and designed the experiments; Performed the experiments; Analyzed and interpreted the data; Contributed reagents, materials, analysis tools or data; Wrote the paper.

Francisco Javier SÁEZ-FERNÁNDEZ: Conceived and designed the experiments; Analyzed and interpreted the data; Wrote the paper.

Andrés J PICAZO-TADEO: Conceived and designed the experiments; Analyzed and interpreted the data; Wrote the paper.

### Funding statement

Andrés J. Picazo-Tadeo aknowledges the financial support from the 10.13039/501100003359Generalitat Valenciana (project PROMETEO 2018/102).

### Data availability statement

The data employed in this research were obtained from Moody's Analytics BankFocus at https://www.bvdinfo.com/en-us/our-products/data/international/bankfocus.

### Declaration of interests statement

The authors declare no conflict of interest.

### Additional information

No additional information is available for this paper.

## Figures and Tables

**Table A1:** Robustness check: Scores of technical efficiency (1 *best*) and potential output loss with ROE.

	Mean	Standard deviation
Weak disposability axiom $\left(1+{\text{β}}^{\text{W}}\right)$	1.639	0.774
Strong disposability axiom $\left(1+{\text{β}}^{\text{S}}\right)$	1.695	0.816
Potential good output loss $\left({\text{β}}^{\text{S}}-{\text{β}}^{\text{W}}\right)$	0.056	0.197

Source: Authors' elaboration.

**Table A2:** Robustness check: Statistical significance of the differences in technical efficiency.(1)

	Kolmogorov-Smirnov test (*KS-statistic*)(2)	Mann-Whitney test (*Z-statistic*)(3)	Simar-Zelenyuk-Li test (*Li-statistic*)(4)
Commercial *versus* investment	0.174 (0.007)^∗∗∗^	-2.165 (0.030)^∗∗^	2.878 (0.002)^∗∗∗^
Domestic *versus* foreign	0.101 (0.198)	-0.356 (0.721)	1.406 (0.079)^∗^
Private *versus* public	0.363 (0.068)^∗^	-1.990 (0.046)^∗∗^	0.163 (0.434)

Source: Authors' elaboration.

∗ means significance at 10%.

(1) *P-values* are in parentheses; ^∗∗∗^ and ^∗∗^ mean significance at 1% and 5%, respectively.

(2) Null hypothesis: the two samples have the same distribution; the exact *p-values* are computed.

(3) Null hypothesis: the two samples are drawn from the same population. *Z-statistic* adjusted for ties.

(4) Original estimates are smoothed using the algorithm II in Simar and Zelenyuk (2006, p.508).

**Table A3:** Robustness check: Managerial efficiency *versus* group efficiency (*1 best*), with ROE.

	Commercial banks		Investment banks	
	Mean	Standard deviation	Mean	Standard deviation
Overall efficiency $\left(1+{\text{β}}^{\text{W}}\right)$	1.664	0.780	1.549	0.746
Managerial efficiency $\left(1+{\text{β}}^{\text{Wg}}\right)$	1.490	0.623	1.174	0.348
Group efficiency $\left(1+{\text{β}}^{\text{W}}\right)/\left(1+{\text{β}}^{\text{Wg}}\right)$	1.110	0.231	1.295	0.425

Source: Authors' elaboration.

**Table A4:** Robustness check: Statistical significance of the differences in overall efficiency and group efficiency(1) between commercial and investment banks, with ROE.

	Kolmogorov-Smirnov test (*KS-statistic*)(2)	Mann-Whitney test (*Z-statistic*)(3)	Simar-Zelenyuk-Li test (*Li-statistic*)(4)
Group efficiency	0.211 (0.001)***	3.101 (0.001)***	0.452 (0.325)

Source: Authors' elaboration.

(1) *P-values* are in parentheses; *** mean significance at 1%.

(2) Null hypothesis: the two samples have the same distribution; exact *p-values* are computed.

(3) Null hypothesis: the two samples are drawn from the same population; *Z-statistic* adjusted for ties.

(4) Original estimates are smoothed using the algorithm II in Simar and Zelenyuk (2006, p.508).

## Footnotes

1 Banking performance could also be assessed with alternative direction vectors; e.g., reducing risk while maintaining the same level of inputs and good outputs, or simultaneously reducing risk and inputs while maintaining or even increasing good outputs.

2 VRS is a common assumption in performance analyses in the banking industry (Barros et al., 2012). In practice, VRS means that each bank is compared against other observed banks of a similar size*—*or linear combinations of the production plan of two or more such observed banks*—*instead of against all banks in the sample. Given the large differences in size that exist in the Brazilian banking industry, in our opinion VRS is the most sensible assumption.

3 In the metatechnology approach we only define the technology with the assumption of weak disposability since it represents the *real world* in which reducing risk is costly.

4 In order to avoid infeasibilities due to banks with DDFs equal to zero with respect to the technology of their own group, both the numerator and the denominator of the metatechnology ratio are formulated as one plus the directional function, either distance or metadistance function.

5 Last access to the data was carried out through https://www.bvdinfo.com/en-us/our-products/data/international/bankfocus on January 14, 2021.

6 Since our sample includes banks performing different activities, an unusually large number of inputs and outputs—including risk—need to be considered in the technology. Moreover, the metatechnology approach requires separately computing scores of performance for particular groups of banks—e.g., commercial and investment—which also reduces the sample size in some programs.

7 The DDFs have been computed using the DJL package in R software.

8 This output loss would amount to an average potential increase in loans by bank and year of 356.8 million constant 2019 $US; and 197.1 million of securities.

9 Brazilian banks normally provide several banking services, including retail services, investment banking services, and brokerage services, as noted in Section 4.2. However, they have been categorized as commercial or investment banks according to their main activity reported to Moody's Analytics.

10 For some of the banks in the sample, information regarding the ownership of capital—domestic *versus* foreign, or private *versus* public—is not provided by our source of data.

11 Jiménez-Hernández et al. (2019b) also employed this approach to assess the differences of efficiency in the management of non-performing loans between cooperative and commercial banks in the Latin American and Caribbean banking industry; see also Jiménez-Hernández et al. (2019a).
